# Efficacy and safety of levosimendan in acute heart failure patients with different body mass index: a multicenter, retrospective, observational real-world study

**DOI:** 10.3389/fcvm.2026.1790085

**Published:** 2026-04-13

**Authors:** Wenjing Liu, Liu Yang, Zhenhuan Chen, Yijie Wu, Hengli Lai

**Affiliations:** 1 Jiangxi Medical College, Nanchang University, Nanchang, China; 2 Department of Cardiology, Jiangxi Provincial People’s Hospital, The First Affiliated Hospital of Nanchang Medical College, Nanchang, China; 3 Department of Cardiology, The First People’s Hospital of Xiushui County, Jiujiang, China

**Keywords:** acute heart failure, body mass index, efficacy and safety, levosimendan, real-world study

## Abstract

**Objective:**

To evaluate the efficacy and safety of levosimendan in acute heart failure (AHF) patients with different body mass index (BMI) using real-world data.

**Methods:**

This retrospective study enrolled 2,398 AHF patients hospitalized at 14 provincial, municipal, and county-level hospitals in Jiangxi Province from March 1, 2023, to March 1, 2025. After excluding 828 cases due to missing data and 60 cases with BMI < 18.5 kg/m^2^, a total of 1,530 patients were included. Patients treated with levosimendan were categorized into three groups: normal weight group (18.5 ≤ BMI < 24, *n* = 535), overweight group (24 ≤ BMI < 28, *n* = 510), and obese group (BMI ≥ 28, *n* = 485). To assess the efficacy and safety of levosimendan in real-world AHF patients overall and across BMI groups, comparisons were made before and 7 days after treatment regarding heart rate, systolic blood pressure (SBP), diastolic blood pressure (DBP), left ventricular ejection fraction (LVEF), NT-proBNP levels, cardiac function classification, respiratory symptoms, pulmonary rales, and adverse reactions.

**Results:**

After 7 days of levosimendan treatment, patients showed significant reductions in SBP and DBP compared to pre-treatment levels (*P* < 0.01). LVEF significantly increased (*P* < 0.01), and NT-proBNP levels significantly decreased (*P* < 0.01). Serum creatinine levels also significantly decreased (*P* < 0.01). Significant improvements were observed in cardiac function classification, respiratory symptoms, and pulmonary rales (all *P* < 0.01). No significant changes were found in heart rate, serum potassium, alanine aminotransferase (ALT), or aspartate aminotransferase (AST) (all *P* > 0.05). Baseline NT-proBNP levels differed significantly among the three groups (normal > overweight > obese, *P* < 0.01), a pattern that persisted after 7 days of treatment (*P* < 0.01). However, there were no significant differences among the groups in the magnitude of NT-proBNP reduction or the proportion of patients achieving a ≥ 30% reduction in NT-proBNP (*P* > 0.05). Compared to the normal weight group, the overweight and obese groups showed significantly greater increases in LVEF after treatment (*P* < 0.01), with the obese group showing a significantly greater increase than the overweight group (*P* < 0.01). The rates of improvement in cardiac function classification and respiratory symptoms were significantly higher in the overweight and obese groups compared to the normal weight group (*P* < 0.01). Although the obese group had slightly higher improvement rates than the overweight group, the difference was not statistically significant (*P* > 0.05). The incidence of hypotension was significantly lower in the overweight and obese groups compared to the normal weight group (*P* < 0.01). The total incidence of adverse reactions was also significantly lower in the overweight and obese groups (*P* < 0.01). Hospital length of stay was significantly shorter in the overweight and obese groups compared to the normal weight group (*P* < 0.05). There were no significant differences among the three groups in heart rate, SBP, DBP, pulmonary rales, serum potassium, ALT, AST, serum creatinine, or other adverse reactions after 7 days of treatment (all *P* > 0.05).

**Conclusions:**

1. In patients with acute heart failure, levosimendan administration was associated with a decreasing trend in plasma NT-proBNP levels, an improvement in left ventricular ejection fraction, and alleviation of cardiac functional impairment and respiratory symptoms. Favorable safety profiles were generally observed during the treatment period. 2. Body mass index showed a negative correlation with NT-proBNP levels, suggesting that patients with higher BMI tend to have relatively lower baseline NT-proBNP levels. 3. In acute heart failure patients stratified by body mass index, levosimendan treatment exhibited between-group variations in clinical parameter improvements and safety profiles. Overweight and obese subgroups demonstrated a tendency toward more pronounced clinical improvement and fewer reported adverse events.

## 1 Introduction

Acute heart failure (AHF) is a severe clinical syndrome characterized by high morbidity, mortality, and economic burden, representing one of the most common causes of hospitalization and death in patients with cardiovascular diseases (1). Levosimendan, a novel inotropic agent, has demonstrated recognized efficacy and safety in clinical studies for both acute and chronic heart failure (2–4). It possesses a unique triple action of enhancing cardiac contractility, vasodilation, and cardioprotection (5, 6). Numerous domestic and international studies have shown that compared to traditional inotropic agents, levosimendan can significantly improve hemodynamics, reduce mortality, and improve prognosis in heart failure patients (7–10).

Obesity is an independent and potent risk factor for heart failure, contributing to the occurrence of cardiovascular risk factors through various mechanisms and promoting the onset, progression, and mortality of cardiovascular diseases (11). Current research has established a “U-shaped” or “J-shaped” curve relationship between BMI and mortality in the field of heart failure, where overweight and obese patients paradoxically exhibit lower mortality risk and better prognosis, a phenomenon known as the “obesity paradox,” though its explanation remains debated (12, 13).

As a new-generation anti-heart failure agent, levosimendan is commonly used in the treatment of acute heart failure. However, its efficacy and safety in acute heart failure patients with different body mass index categories remain unreported. Moreover, real-world studies on the efficacy and safety of levosimendan for acute heart failure are limited in number, with existing research primarily focusing on specific populations (e.g., the elderly and patients with renal insufficiency). Previous investigations have predominantly been randomized controlled trials. As a multicenter real-world study conducted in Jiangxi, China, involving 1,530 patients, the present research demonstrates notable advantages in sample size and clinical representativeness. It provides valuable evidence-based support for the practical application of levosimendan in the management of acute heart failure. Its focus on the Chinese population and multidimensional efficacy assessment address existing gaps in the current literature.

## 2 Materials and methods

### 2.1 Study Population

This was a multicenter, retrospective, observational study. A total of 2,398 AHF patients hospitalized at 14 provincial, municipal, and county-level hospitals in Jiangxi Province from March 1, 2023, to March 1, 2025, were initially screened. After excluding 828 cases due to missing data and 60 cases with BMI <18.5 kg/m², 1,530 patients were finally included.

Inclusion criteria: (1) Age ≥18 years, gender-neutral; (2) Patients diagnosed with AHF according to diagnostic criteria (Combining medical history, clinical signs and symptoms, natriuretic peptide levels, and cardiac imaging studies, NT-proBNP >450 pg/mL for age <50, >900 pg/mL for age 50–75, >1,800 pg/mL for age >75) (14); (3) Patients and their families agreed to levosimendan treatment; (4) Patients without severe hypotension risk (SBP <80 mmHg or cardiogenic shock) (15, 16); (5) Patients assessed by investigators as suitable for levosimendan treatment during hospitalization.

Exclusion criteria: (1) Patients with acute cerebrovascular events; (2) Patients allergic to levosimendan or any excipients; (3) Patients with mechanical obstructive diseases significantly affecting ventricular filling or/and ejection; (4) Patients with severe liver or renal impairment (creatinine clearance rate <30 mL/min); (5) Patients with severe hypotension and tachycardia (SBP <80 mmHg, heart rate >120 bpm); (6) Patients with a history of Torsades de Pointes (TdP); (7) Patients deemed unsuitable for inclusion by investigators.

This study was approved by the Medical Ethics Committee of Jiangxi Provincial People's Hospital (Ethics Approval No.: 2024-053 IIT, Approval Date: May 7, 2024). All patients provided informed consent.

### 2.2 Research Methods

#### 2.2.1 Treatment Regimen

After admission, patients received levosimendan intravenously via micro-infusion pump at a dose of 0.05–0.2 μg/(kg·min) for 24 h without a loading dose. Blood pressure was closely monitored during treatment, and the dose was adjusted based on clinical presentation. If blood pressure decreased further, dopamine or norepinephrine was used for support. If blood pressure remained difficult to maintain despite vasopressors, levosimendan was discontinued. Other treatments, including ACEI/ARBs, diuretics, beta-blockers, and SGLT2 inhibitors, were administered according to the patient's condition.

#### 2.2.2 BMI measurement method

In this study, height and body weight data used for BMI calculation were obtained by trained nursing staff at the time of the patient's initial assessment in the emergency department or inpatient unit upon admission. Measurements were taken using a standard calibrated weighing scale prior to the administration of any intravenous diuretic therapy.

#### 2.2.3 LVEF measurement method

Transthoracic echocardiography was performed with the patient in the left lateral decubitus or supine position. The chest was fully exposed, and a phased-array transducer was positioned at the left parasternal window (3rd-4th intercostal space) to obtain the parasternal long-axis view. Transducer angulation and imaging depth were optimized to clearly visualize the left ventricle, interventricular septum, left atrium, and aorta. Comprehensive cardiac chamber quantification and functional assessment were performed from multiple acoustic windows including the apical four-chamber view and parasternal long-axis plane to measure cardiac chamber dimensions and evaluate systolic and diastolic function (17).

#### 2.2.4 Grouping

Among the study subjects meeting the inclusion and exclusion criteria, those treated with levosimendan were categorized as follows: normal weight group (18.5 ≤ BMI < 24, *n* = 535), overweight group (24 ≤ BMI < 28, *n* = 510), and obese group (BMI ≥ 28, *n* = 485) (18).

Demographic data, medical history, medication history, NYHA functional class, laboratory tests, and echocardiographic parameters from the first admission were collected. All patients underwent continuous ECG and blood pressure monitoring during hospitalization, recording changes in blood pressure, heart rate, and rhythm.

### 2.3 Outcome measures

**Primary Efficacy Endpoints:**

1. Symptom change: Dyspnea was assessed in all patients before and after treatment using a scoring system: no dyspnea (0 point); exertional dyspnea (1 points); paroxysmal nocturnal dyspnea (2 points); orthopnea (3 points). Improvement was rated by the score difference: marked improvement (2–3 point decrease); improvement (1 point decrease); no change (0 point change); deterioration (decrease of −1 point or less). Marked improvement and improvement were counted towards the total effective rate. Total effective rate (%) = (Number of marked improvement cases + Number of improvement cases)/Total number of cases ×  100% (19).
2. Change in cardiac function classification: Cardiac function was assessed before and after treatment in all patients. The Killip classification was used for acute myocardial infarction patients, and the NYHA classification for others. Efficacy criteria: Improvement by ≥2 classes was marked effectiveness; improvement by 1 class was effectiveness; improvement <1 class or worsening was ineffective. Marked effectiveness and effectiveness were included in the total effective rate. Total effective rate (%) = (Number of marked effectiveness cases + Number of effectiveness cases)/Total number of cases ×  100% (20).
3. Laboratory indicators: Change in NT-proBNP levels: Plasma NT-proBNP was measured before and after treatment in all patients. A reduction in NT-proBNP ≥30% from baseline after AHF treatment indicated effective therapy (21).

**Secondary Efficacy Endpoints:**

1. Length of hospital stay;
2. Changes in physical signs: (1) Changes in blood pressure; (2) pulmonary rales after 7 days of treatment.
3. Echocardiogram examination:Changes in LVEF.
4. Laboratory indicators: (1) Liver and kidney function: Changes in SCr, ALT, AST before and after treatment.

**Safety Evaluation Endpoints:**

1. Laboratory indicators: Electrolytes: Changes in serum K+ concentration before and after treatment;
2. Occurrence of adverse reactions: (1) Changes in physical signs: a. Hypotension: SBP < 80 mmHg, regardless of symptoms (15, 16); b.Tachycardia: Heart rate > 120 bpm, regardless of symptoms (15, 16); (2)Laboratory indicators: Hypokalemia: Serum K+ <3.5 mmol/L (22);
3. Other adverse reactions: Cardiac arrest, myocardial infarction, atrial fibrillation, worsening heart failure, dizziness, acute cerebral infarction, or other symptoms.

### 2.4 Statistical analysis

Statistical analysis was performed using SPSS 27.0 software. NT-proBNP values underwent natural logarithmic transformation. Normally distributed continuous data are presented as mean ± standard deviation (SD), while categorical data are presented as number (%). Comparisons within groups before and after treatment were performed using paired sample *t*-tests. Intergroup comparisons were conducted using one-way ANOVA, with *post-hoc* multiple comparisons for pairwise analysis. Differences in categorical data between groups were compared using the Chi-square test or Fisher’s exact test. The correlation between BMI and NT-proBNP was analyzed using Spearman's rank correlation test. All tests were two-sided, with *P* < 0.05 considered statistically significant.

## 3 Results

### 3.1 Disposition of subjects

Among the 2,398 initially enrolled patients, 828 were excluded due to missing data, and 60 underweight patients (BMI <18.5 kg/m²) were excluded due to insufficient sample size. Finally, 1,530 patients completed the study: 535 in the normal weight group, 510 in the overweight group, and 485 in the obese group.

### 3.2 Comparison of patient indicators before and after levosimendan treatment

**Efficacy Endpoints:** A significant difference was found between pre-treatment NT-proBNP ln[(8.75 ± 0.55) pg/mL] and post-7-day treatment NT-proBNP ln[(7.79 ± 0.78) pg/mL] (*P* < 0.01). Significant improvements were observed in cardiac function classification, respiratory symptoms, and pulmonary rales after 7 days of treatment (all *P* < 0.01). LVEF increased from (41.4 ± 7.7)% pre-treatment to (45.0 ± 6.6)% post-treatment (*P* < 0.01). SBP decreased from (125.0 ± 21.7) mmHg to (114.8 ± 16.0) mmHg (*P* < 0.01). DBP decreased from (76.1 ± 15.5) mmHg to (70.0 ± 10.7) mmHg (*P* < 0.01). No significant change was observed in heart rate (*P* > 0.05).Serum creatinine decreased from (94.7 ± 26.1) μmol/L pre-treatment to (85.7 ± 22.4) μmol/L post-treatment (*P* < 0.01). No significant changes were found in serum potassium, ALT, or AST (all *P* > 0.05). Details are shown in Tables 1, 2.

**Table 1:** Comparison of vital signs, laboratory parameters, and left ventricular ejection fraction in the overall study population.

Parameter	At admission	After 7 Days of Treatment	Change	*P* value
Heart Rate (beats/min)	86.4 ± 17.7	86.1 ± 17.1	0.3 ± 17.0	0.519
Systolic Blood Pressure（mmHg）	125.0 ± 21.7	114.8 ± 16.0	10.2 ± 17.1	<0.01
Diastolic Blood Pressure（mmHg）	76.1 ± 15.5	70.0 ± 10.7	6.1 ± 12.1	<0.01
LVEF（%）	41.4 ± 7.7	45.0 ± 6.6	−3.6 ± 4.9	<0.01
lnNT-proBNP（pg/mL）	8.75 ± 0.55	7.79 ± 0.78	0.96 ± 0.72	<0.01
Serum Potassium（mmol/L）	4.14 ± 0.58	4.12 ± 0.36	0.02 ± 0.6	0.241
Alanine Aminotransferase（U/L）	30.5 ± 17.4	29.8 ± 15.2	0.7 ± 15.4	0.098
Aspartate Aminotransferase（U/L）	27.12 ± 13.9	27.15 ± 13.7	0.03 ± 13.0	0.94
Serum Creatinine（umol/L）	94.7 ± 26.1	85.7 ± 22.4	9.0 ± 21.5	<0.01

**Table 2:** Improvement in cardiac function, respiratory symptoms, and pulmonary rales in the overall study population.

Parameter	At admission	After 7 Days of Treatment	*P* value	Number of effective cases (percentage)
Cardiac Function Classification			<0.01	1,244（81.3）
I	0	205		
II	219	1,067		
III	1,026	189		
IV	285	69		
Respiratory Symptoms			<0.01	1,245（81.4）
No Dyspnea	0	904		
Exertional Dyspnea	832	474		
Paroxysmal Nocturnal Dyspnea	427	93		
Orthopnea	271	59		
Pulmonary rales			<0.01	875（90.5）
Present	966	91		
Absent	564	1,439		

### 3.3 Comparison of baseline characteristics among the three groups

Baseline NT-proBNP levels differed significantly among the three groups (*P* < 0.01), with the order being normal > overweight > obese. No significant differences were found among the three groups in age, gender, medical history, medication history, heart rate, SBP, DBP, LVEF, serum creatinine, cardiac function classification, respiratory symptoms, or pulmonary rales (all *P* > 0.05). Details are shown in Table 3.

**Table 3:** Comparison of baseline characteristics among the three patient groups.

Parameter	Normal group	Overweight group	Obese group	*P* value
Age (years, ± SD)	66 ± 11.5	66.2 ± 11.2	65.2 ± 10.6	0.374
Male	389（72.71）	371（72.75）	351（72.37）	0.989
Comorbidities [*n* (%)]				
Hypertension	281（52.52）	288（56.47）	275（56.7）	0.313
Diabetes mellitus	133（24.86）	136（26.67）	146（30.1）	0.164
Coronary artery disease	188（35.14）	161（31.57）	173（35.67）	0.326
Chronic heart failure	480（89.72）	474（92.94）	434（89.48）	0.105
Medication history [*n* (%)]				
ACEI/ARB	253（47.29）	265（51.96）	227（46.8）	0.193
β-Adrenergic receptor blocker	207（38.69）	187（36.67）	190（39.18）	0.685
Diuretics	485（90.65）	475（93.14）	446（91.96）	0.339
SGLT2	158（29.53）	156（30.59）	153（31.55）	0.784
Vital signs				
Heart Rate (beats/min)	86 ± 17.1	86.9 ± 18.3	86.1 ± 17.9	0.623
Systolic Blood Pressu（mmHg）	124.1 ± 21.8	125 ± 20.9	125.9 ± 22.5	0.419
Diastolic Blood Pressure（mmHg）	76.3 ± 16.2	75.8 ± 14.7	76.1 ± 15.3	0.887
LVEF（%, ± SD）	41.5 ± 8.19	41.7 ± 7.7	41.1 ± 7.1	0.311
Laboratory examinations on admission				
lnNT-proBNP(pg/mL, ± SD)	8.87 ± 0.416	8.74 ± 0.66^a^	8.64 ± 0.53^a^^,^^b^	<0.01
Serum Creatinine（umol/L, ± SD）	96.4 ± 27.2	94.3 ± 23.5	93.3 ± 27.3	0.159
Heart function classification on admission[*n*(%)]				0.537
II	76（14.21）	71（13.92）	72（14.85）	
III	347（64.86）	350（68.63）	329（67.84）	
IV	112（20.93）	89（17.45）	84（17.32）	
Respiratory symptoms on admission[*n*(%)]				0.843
Exertional Dyspnea	294（54.95）	278（54.51）	260（53.61）	
Paroxysmal Nocturnal Dyspnea	144（26.92）	148（29.02）	135（27.84）	
Orthopnea	97（18.13）	84（16.47）	90（18.55）	

^a^ *P* < 0.0 5; compared with normal BMI group.

^b^ *P* < 0.05; compared with overweight group.

### 3.4 Comparison of outcome measures among the three groups

**Efficacy Endpoints:** Baseline NT-proBNP levels differed significantly (normal > overweight > obese, *P* < 0.01), and this difference persisted after 7 days of treatment (*P* < 0.01). No significant differences were found among the three groups in the magnitude of NT-proBNP reduction or the proportion of patients achieving a ≥30% reduction in NT-proBNP (*P* > 0.05).

Compared to the normal weight group, the overweight and obese groups showed significantly greater increases in LVEF after treatment (*P* < 0.01). The obese group showed a significantly greater increase in LVEF than the overweight group (*P* < 0.01).

The rates of improvement in cardiac function classification and respiratory symptoms were significantly higher in the overweight and obese groups compared to the normal weight group (*P* < 0.01). Although the obese group had slightly higher rates than the overweight group, the difference was not statistically significant (*P* > 0.05).

Hospital length of stay was significantly shorter in the overweight and obese groups compared to the normal weight group (*P* < 0.05). Although the obese group had a slightly shorter stay than the overweight group, the difference was not statistically significant (*P* > 0.05).

No significant differences were found among the three groups in ALT, AST, serum creatinine (*P* > 0.05).

Safety Endpoints: The incidence of hypotension was significantly lower in the overweight and obese groups compared to the normal weight group (*P* < 0.01). Although the obese group had a slightly lower incidence than the overweight group, the difference was not statistically significant (*P* > 0.05).

The total incidence of adverse reactions was significantly lower in the overweight and obese groups compared to the normal weight group (*P* < 0.01). Although the obese group had a slightly higher total incidence than the overweight group, the difference was not statistically significant (*P* > 0.05).

**Figure 1:**
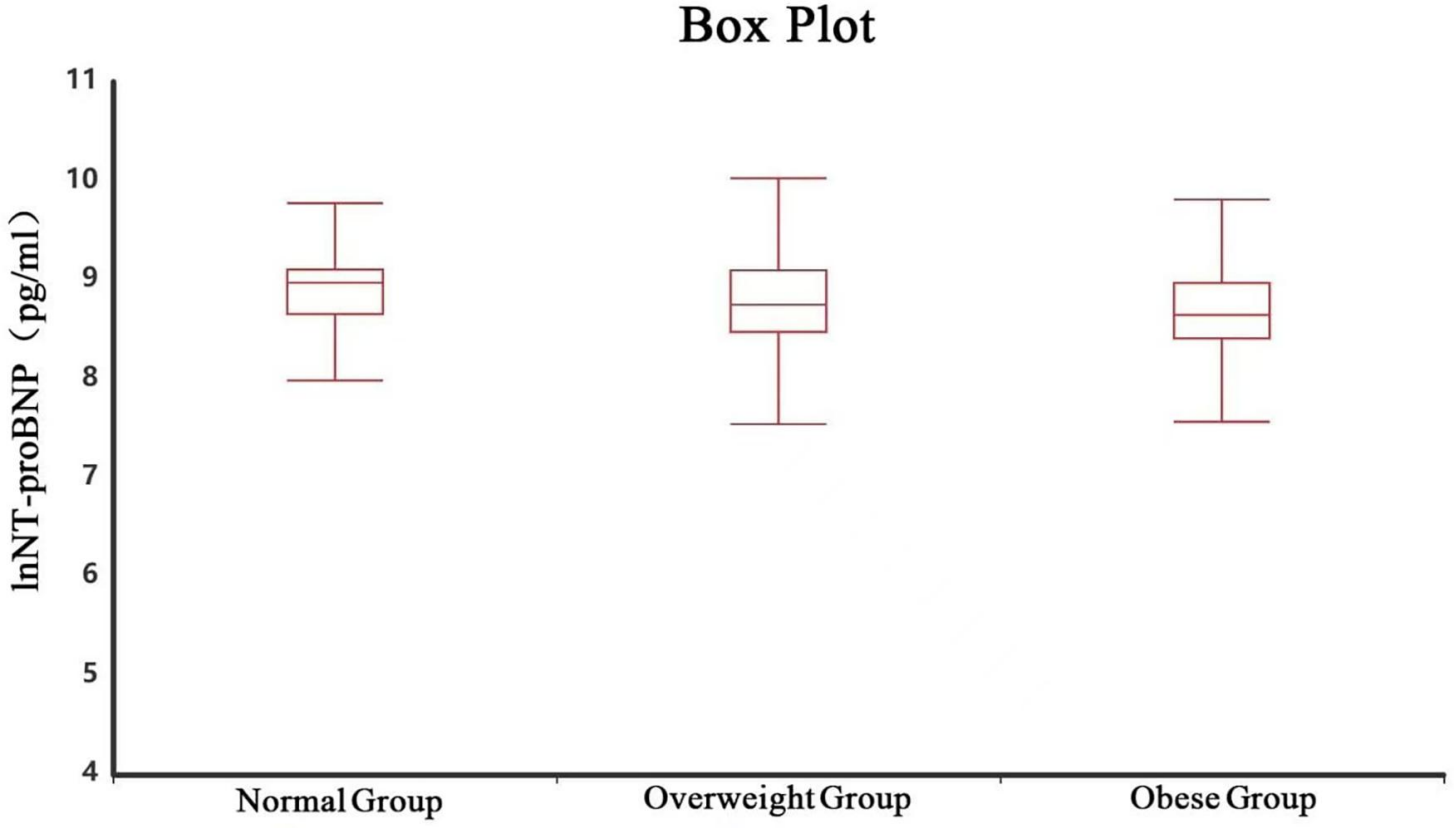
Distribution of BMI and lnNT-proBNP.

No significant differences were found among the three groups in serum potassium, or other adverse reactions after 7 days of treatment (all *P* > 0.05). Details are shown in Tables 4–6 and Figure 1.

**Table 4:** Comparison of vital signs, laboratory parameters, left ventricular ejection fraction, and hospital stay among the normal weight, overweight, and obesity groups.

Parameter	Normal group	Overweight group	Obese group	*P* value
Heart rate at admission (beats per minute)	86 ± 17.1	86.9 ± 18.3	86.1 ± 17.9	0.623
Heart rate after 7 days of treatment (beats per minute)	85.8 ± 17.2	86 ± 16.6	86.4 ± 17.5	0.875
Change	0.2 ± 16.8	0.9 ± 18.0	−0.3 ± 16.3	0.498
Systolic blood pressure at admission (mmHg)	124.1 ± 21.8	125 ± 20.9	125.9 ± 22.5	0.419
Systolic blood pressure after 7 days of treatment (mmHg)	113.5 ± 16.4	115.2 ± 15.2	115.7 ± 16.1	0.072
Change	10.6 ± 16.1	9.8 ± 17.5	10.2 ± 17.8	0.728
Diastolic blood pressure at admission (mmHg)	76.3 ± 16.2	75.8 ± 14.7	76.1 ± 15.3	0.887
Diastolic blood pressure after 7 days of treatment (mmHg)	70.3 ± 10.5	69.8 ± 10.6	69.9 ± 11	0.549
Change	6.0 ± 13.3	6.0 ± 11.1	6.2 ± 11.7	0.797
Left ventricular ejection fraction at admission (%)	41.5 ± 8.19	41.7 ± 7.7	41.1 ± 7.1	0.311
Left ventricular ejection fraction after 7 days of treatment (%)	44.1 ± 6.7	45.3 ± 7.2^a^	45.7 ± 5.8^a^	<0.01
Change	2.6 ± 4.86	3.6 ± 4.96^a^	4.6 ± 4.54^a^^,^^b^	<0.01
lnNT-ProBNP（pg/mL） at admission	8.87 ± 0.416	8.74 ± 0.66^a^	8.64 ± 0.53^a^^,^^b^	<0.01
after 7 days of treatment	7.93 ± 0.75	7.79 ± 0.86^a^	7.66 ± 0.71^a^^,^^b^	<0.01
Change	0.94 ± 0.59	0.95 ± 0.64	0.98 ± 0.63	0.5
Number of effective cases	446（83.3）	418（81.9）	403（83.0）	0.818
Serum potassium at admission(mmol/L)	4.13 ± 0.62	4.14 ± 0.56	4.15 ± 0.55	0.878
Serum potassium after 7 days of treatment (mmol/L)	4.1 ± 0.37	4.13 ± 0.35	4.14 ± 0.36	0.195
Change	0.03 ± 0.64	0.01 ± 0.59	0.01 ± 0.57	0.687
Alanine aminotransferase at admission (U/L)	30.5 ± 17.5	29.7 ± 17.9	31.3 ± 16.5	0.354
Alanine aminotransferase after 7 days of treatment (Ul/L)	29.1 ± 15.6	29.8 ± 14.6	30.7 ± 15.3	0.23
Change	1.4 ± 15.8	−0.1 ± 14.8	0.6 ± 15.5	0.321
Aspartate aminotransferase at admission (U/L)	27.1 ± 12.7	27.4 ± 14.3	26.8 ± 14.9	0.818
Aspartate aminotransferase after 7 days of treatment (Ul/L)	27.6 ± 11.9	26.2 ± 11.8	27.5 ± 17.1	0.194
Change	−0.5 ± 9.0	1.2 ± 12.1	−0.7 ± 14.8	0.055
Serum creatinine at admission (*μ*mol/L)	96.4 ± 27.2	94.3 ± 23.5	93.3 ± 27.3	0.159
Serum creatinine after 7 days of treatment (μmol/L)	87.1 ± 24.9	85.7 ± 20.1	84.3 ± 21.6	0.12
Change	9.3 ± 23.6	8.6 ± 16.4	9.0 ± 21.5	0.864

^a^ *P* < 0.05; compared to the normal BMI group.

^b^ *P* < 0.05; compared to the overweight group.

**Table 5:** Improvement in cardiac function, respiratory symptoms, and pulmonary rales among the normal weight, overweight, and obesity groups.

Parameter	Normal group	Overweight group	Obese group	*P* value
Heart function classification on admission				0.537
II	76	71	72	
III	347	350	329	
IV	112	89	84	
Heart function classification after 7 days of treatment				0.06
I	72	64	69	
II	357	361	349	
III	71	69	49	
IV	35	16	18	
Number of cases with effective heart function improvement (percentage)	413（77.2）	424（83.1)^a^	407（83.9)^a^	<0.01
Respiratory symptoms on admission				0.843
No Dyspnea	0	0	0	
Exertional Dyspnea	294	278	256	
Paroxysmal Nocturnal Dyspnea	144	148	139	
Orthopnea	97	84	90	
Respiratory symptoms after 7 days of treatment				0.069
No Dyspnea	303	298	303	
Exertional Dyspnea	165	169	140	
Paroxysmal Nocturnal Dyspnea	37	31	25	
Orthopnea	30	12	17	
Number of cases with effective improvement in respiratory symptoms (percentage)	417（77.9）	423（83)^a^	405（84)^a^	<0.01
Pulmonary rales on admission				0.861
Present	335	320	311	
Absent	200	190	174	
Pulmonary rales after 7 days of treatment				0.528
Present	28	35	28	
Absent	507	475	457	
Number of cases with effective improvement in pulmonary rales (percentage)	307（91.6）	285（89.1）	283（91）	0.504

Improvement in cardiac function is defined as an increase of ≥1 grade; respiratory symptoms are considered improved if the score decreases by ≥1 point; improvement in pulmonary rales is defined as the absence of rales where they were previously present.

^a^ *P* < 0.05; compared to the normal BMI group.

^b^ *P* < 0.05; compared to the overweight group.

**Table 6:** Comparison of adverse events among the normal weight, overweight, and obesity groups.

Parameter	Normal group	Overweight group	Obese group	*P* value
Length of hospital stay (days)	12.9 ± 9.1	11.9 ± 7.7^a^	11.4 ± 7.8^a^	<0.01
Hypotension (number of cases and percentage)	35（6.5）	17（3.3)^a^	15（3.1)^a^	0.01
Hypokalemia (number of cases and percentage)	28（5.2）	20（4）	21（4）	0.578
Tachycardia (number of cases and percentage)	24（4.5）	19（3.7）	24（4.9）	0.634
Atrial fibrillation	4（0.7）	2（0.4）	1（0.2）	0.425
Worsening heart failure	2（0.4）	1（0.2）	2（0.4）	0.813
Dizziness	2（0.4）	1（0.2）	0（0）	0.405
Total adverse reactions (number of cases and percentage)	95（17.7）	60（11.7)^a^	63（12.7)^a^	0.014

Hypotension is defined as a systolic blood pressure below 80 mmHg; tachycardia is defined as a heart rate exceeding 120 beats per minute.

^a^ *P* < 0.05; compared to the normal BMI group.

^b^ *P* < 0.05; compared to the overweight group.

## 4 Discussion

Levosimendan, a novel inotropic agent, possesses a unique triple action of enhancing cardiac contractility, vasodilation, and cardioprotection: (1) Calcium sensitization: It binds to cardiac troponin C (cTnC) in a Ca²^+^-dependent manner, enhancing myocardial contraction without increasing intracellular Ca²^+^ concentration, impairing diastolic function, activating the sympathetic nervous system, or increasing myocardial oxygen consumption. (2) Vasodilation: It opens ATP-sensitive K^+^ channels in vascular smooth muscle cells, leading to vasodilation. This reduces cardiac preload and afterload, dilates both large and small vessels, reduces coronary vascular resistance, improves organ microcirculation, and increases coronary blood flow. (3) Cardioprotection: It opens mitochondrial KATP channels, exerting an ischemic preconditioning-like myocardial protective effect. This improves myocardial tissue perfusion, reduces infarct size (anti-ischemic effect), enhances cardiomyocyte tolerance to hypoxia, and prevents ischemia-reperfusion injury (6).

In clinical research, randomized controlled trials (RCTs) are often regarded as the “gold standard” for evidence. They strictly control inclusion/exclusion criteria and standardize interventions, leading to an “idealized” patient population that may not represent real-world clinical practice. Real-world studies, based on data generated in actual healthcare settings, encompass patients of various ages, comorbidities, and treatment adherence, reflecting the true clinical environment and complementing the limitations of traditional RCTs. While several RCTs have confirmed the efficacy and safety of levosimendan in AHF patients (2–4), evidence from real-world studies is still lacking. This multicenter study in Jiangxi Province observed AHF patients receiving levosimendan across different settings, collecting data on clinical symptoms, relevant indicators, and adverse reactions. It reaffirms the efficacy and safety of levosimendan for AHF in a real-world context. Furthermore, this study is the first to evaluate the efficacy and safety of levosimendan in AHF patients with different BMI using real-world data, providing practical evidence to support rational clinical use and AHF treatment.

NT-proBNP is currently the most valuable biomarker for diagnosing heart failure and can be used for diagnosing various types of heart failure (23). Some studies have indicated a significant negative correlation between NT-proBNP and BMI in heart failure patients; the higher the BMI, the lower the NT-proBNP level (24, 25). This study found significant differences in baseline NT-proBNP levels among the three AHF groups (normal > overweight > obese), with higher BMI associated with lower NT-proBNP, consistent with the aforementioned studies. The mechanism underlying the negative correlation between BMI and plasma NT-proBNP levels may be related to increased total body fluid volume in individuals with high BMI, leading to a dilutional decrease in plasma NT-proBNP concentration. Additionally, obesity is often associated with insulin resistance, and insulin directly inhibits myocardial pro-BNP mRNA expression, reducing NT-proBNP synthesis and secretion (26). Importantly, despite substantial differences in baseline NT-proBNP levels among AHF patients with different BMI, levosimendan treatment resulted in similar magnitudes of NT-proBNP reduction and similar response rates across groups. These findings further confirm the effectiveness of levosimendan in treating AHF patients.

Levosimendan enhances myocardial contractility via Ca²^+^ sensitization and dilates blood vessels by opening vascular smooth muscle KATP channels, reducing cardiac afterload, thereby improving LVEF in heart failure patients. The results of this study show that among different BMI groups treated with levosimendan, compared to the normal weight group, the overweight and obese groups exhibited significantly greater increases in LVEF after treatment. Furthermore, the obese group showed a significantly greater increase than the overweight group. This suggests a hierarchy in LVEF improvement: obese > overweight > normal, indicating that BMI may be associated with the degree of LVEF improvement. Some studies suggest that higher BMI is closely related to LVEF recovery and improvement, and BMI is an effective predictor of LVEF improvement (27). The mechanism may be related to higher muscle mass and metabolic reserves in the form of adipose tissue in patients with high BMI, allowing them to better cope with the catabolic state of heart failure (27). Moreover, patients with higher BMI have greater blood volume and higher preload/afterload. Levosimendan's potent vasodilatory effects might enable these patients to achieve greater increases in LVEF more readily. Studies have shown that higher LVEF is associated with improved survival in HFrEF patients, and the better prognosis and survival observed in obese individuals correlate with parallel and significant increases in LVEF (28, 29), suggesting that overweight and obese heart failure patients may have better prognosis and survival.

This study found that among different BMI groups treated with levosimendan, overweight and obese AHF patients had significantly higher rates of improvement in cardiac function and respiratory symptoms, as well as significantly shorter hospital stays, compared to the normal weight group. This suggests that overweight and obese AHF patients may have better therapeutic efficacy and prognosis than normal-weight heart failure patients. Possible mechanisms include (30–34): (1) AHF is a hypermetabolic, high-energy-demand state. Overweight and obese patients, with greater muscle and fat mass, may possess larger energy and metabolic reserves, enabling them to better withstand the energetic demands and stress of acute heart failure decompensation and respond better to inotropic agents. (2) Overweight and obese patients have more adipose tissue, which chronically releases inflammatory cytokines, placing them in a state of chronic, low-grade inflammation. Levosimendan possesses anti-inflammatory and endothelial protective properties, improving vascular function. Its anti-inflammatory effects might provide greater relative value in overweight/obese patients, more effectively protecting the myocardium. (3) Overweight and obese patients have larger blood volumes and higher cardiac loads (preload and afterload). Levosimendan's potent vasodilatory effects can effectively reduce these loads, rapidly alleviating pulmonary congestion and dyspnea. (4) Overweight and obese patients, with more adipose tissue, often have comorbid sleep apnea syndrome and pulmonary hypertension, increasing right ventricular load. Several studies have confirmed that levosimendan can effectively reduce pulmonary artery pressure, enhance right ventricular contractility, and provide protection to the right heart.

Foreign studies have indicated that compared to patients with normal BMI, overweight or obese heart failure patients are less likely to develop hypotension (35). This study found that normal-weight AHF patients treated with levosimendan had a higher incidence of hypotension compared to overweight and obese patients. The incidence was 3.2% higher than in the overweight group and 3.4% higher than in the obese group. Possible mechanisms include: (1) Levosimendan is a lipophilic drug with high plasma protein binding. Overweight and obese patients, with more adipose tissue, have a larger volume of distribution, leading to relatively lower peak plasma concentrations, which may help mitigate the risk of vasodilation-related hypotension. (2) Studies suggest that patients with renal dysfunction have poorer tolerance to hypotension, and the risk of hypotension increases with decreasing eGFR (36, 37). Overweight and obese patients, characterized by high circulatory volume and glomerular hyperfiltration, may have compensatory early increases in eGFR compared to lower-weight individuals, potentially exhibiting better renal function and relatively lower hypotension risk.

In summary, this study further validated the efficacy and safety of levosimendan treatment in patients with acute heart failure using real-world data. levosimendan therapy was associated with reduced NT-proBNP levels, improved cardiac function, and relief of respiratory symptoms in acute heart failure patients, with no significant impact on hepatic or renal function and a low incidence of adverse reactions. Additionally, this study observed a negative correlation between BMI and baseline NT-proBNP levels, indicating that patients with higher BMI exhibited lower NT-proBNP levels. Furthermore, the results indicate that the effects of levosimendan on cardiac function, respiratory symptoms, biomarkers, and safety showed differential trends across acute heart failure patients with varying body mass indexes. More favorable treatment responses and lower adverse event rates were observed in overweight and obese patients. This study may provide more precise guidance for the clinical use of levosimendan based on different BMI classifications.

## 5 Limitations

The retrospective observational design, lacking a parallel control group, limits the strength of causal inference and makes it difficult to avoid the influence of confounding factors. Treatment protocols (dose, duration, concomitant medications, etc.) might have varied across different centers, affecting the homogeneity of results. Some subjective assessment indicators (e.g., cardiac function assessment, dyspnea scoring, auscultation of pulmonary rales) are susceptible to bias. Furthermore, BMI has inherent limitations; it cannot distinguish between muscle and fat distribution. Other obesity indicators such as waist-to-hip ratio, waist-to-height ratio, weight-adjusted waist circumference index, or visceral fat index might better reflect body fat distribution. This study grouped patients based on BMI measured at admission, a metric potentially influenced by acute fluid overload and diuretic medications, which may not accurately reflect patients' chronic or stable-phase weight status. Future prospective studies that repeat weight measurements when patients reach a clinically defined “dry weight” state will more precisely define the relationship between obesity and prognosis. Image quality challenges arising from acoustic window limitations remain a recognized constraint in LVEF assessment. This may introduce measurement errors and potentially affect the precision of observing minute variations in LVEF. Future studies may consider employing imaging techniques less affected by acoustic windows—such as cardiac magnetic resonance imaging—for further validation. This study did not establish underweight or obesity subtype groups. Future prospective randomized controlled trials or propensityscore matching studies are recommended for further validation.

## Data Availability

The original contributions presented in the study are included in the article/Supplementary Material, further inquiries can be directed to the corresponding author.
